# Renal Leiomyosarcoma: Unraveling the Mysteries of a Rare Malignancy

**DOI:** 10.7759/cureus.45476

**Published:** 2023-09-18

**Authors:** FNU Poombal, Muhammad Ahsan, Rida Noor, Saira Nasir, Anam Khan

**Affiliations:** 1 Pathology, Nishtar Medical University, Multan, PAK; 2 Histopathology, Chughtai Institute of Pathology, Lahore, PAK; 3 Pathology, Faisalabad Medical University, Faisalabad, PAK; 4 Pathology, Chughtai Institute of Pathology, Lahore, PAK

**Keywords:** muscle neoplasm, nephrectomy, leiomyosarcoma, sarcoma, kidney

## Abstract

Primary leiomyosarcoma is a rare malignant kidney tumor. The diagnosis of this disease is usually made on the basis of histological examination because it lacks specific clinical or radiological characteristics. Differentiation between leiomyosarcoma and sarcomatoid renal cell carcinoma can be challenging because spindle cell morphology is observed in both tumors. Therefore, caution should be exercised when making a diagnosis of primary renal leiomyosarcoma. Both renal sarcoma and sarcomatoid renal cell carcinoma have a worse prognosis, and nephrectomy is the treatment of choice in locally resectable tumors. An example of such a tumor is discussed in relation to its diagnostic challenges. We report a case of a 35-year-old female who presented with a left renal mass. A left radical nephrectomy was performed, and a firm, tan-white, lobulated tumor (14x8x7.5 cm) was present on gross examination. A histological diagnosis of high-grade leiomyosarcoma was made on the basis of histology, positivity for caldesmon and desmin, and negative cytokeratin immunostaining. Sarcomatoid renal cell carcinoma was ruled out based on morphological findings after extensive sampling of the tumor along with negativity for CK, CD-10, and carbonic anhydrase IX immunostaining.

## Introduction

In the kidney, leiomyosarcoma is an uncommon and aggressive smooth muscle tumor that originates in the renal pelvis and intrarenal vessels. It represents a mere 0.1% [1,2] of all primary kidney tumors and demonstrates a predilection for females [1] between the ages of 50 and 60. X chromosome genes may contribute to the higher incidence among women [3]. Leiomyosarcomas, however, presents in a similar manner to other renal tumors, often manifesting as hematuria and abdominal pain, which complicates its differentiation from renal cell carcinoma [4]. One noteworthy finding in leiomyosarcoma cases is the finding that 55% of patients have tumors that extend directly beyond the kidney capsule [2], indicating its aggressive nature. In addition, a thrombus extended into the inferior vena cava, indicating a high potential for the spread of the tumor [5]. Alarmingly, 90% of patients eventually develop distant metastases, contributing to the unfavorable prognosis associated with this malignancy [2]. Ultimately, 75% of individuals affected by renal leiomyosarcoma succumb to the burden of their tumor [2]. Given the grave outcomes and limited treatment options, there is an urgent need for additional research to delve into this rare malignancy and to uncover more effective therapeutic strategies.

## Case presentation

**Figure 1:**
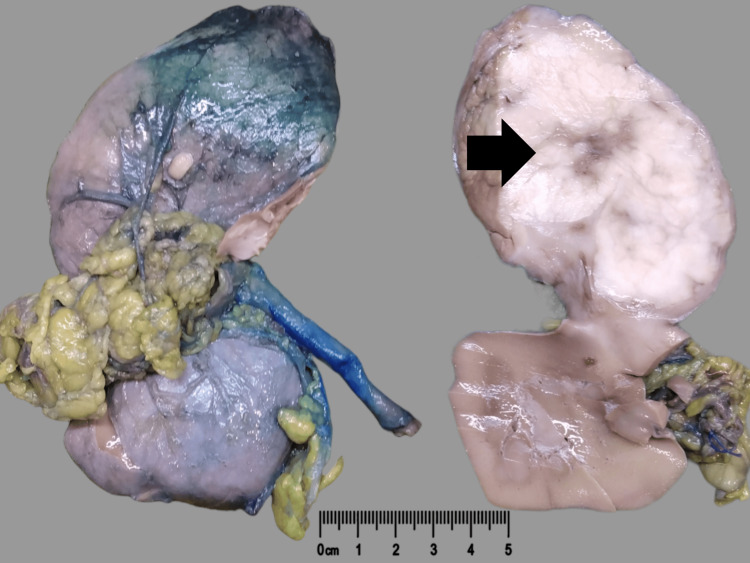
Well-circumscribed, firm white tumor (black arrow) in the kidney

In this report, we describe the case of a 35-year-old female with hematuria and painful urination. Clinical and radiological findings were suggestive of renal cell carcinoma. A left radical nephrectomy was performed on the patient, during which the left renal mass was successfully removed, and a surgical specimen was referred to our laboratory. The kidney capsule was intact. Gross examination showed a firm tan-white, lobulated tumor (14x8x7.5 cm) involving the upper pole and mid-part of the kidney (Figure 1). Areas of necrosis (greater than 50%) were also observed.

**Figure 2:**
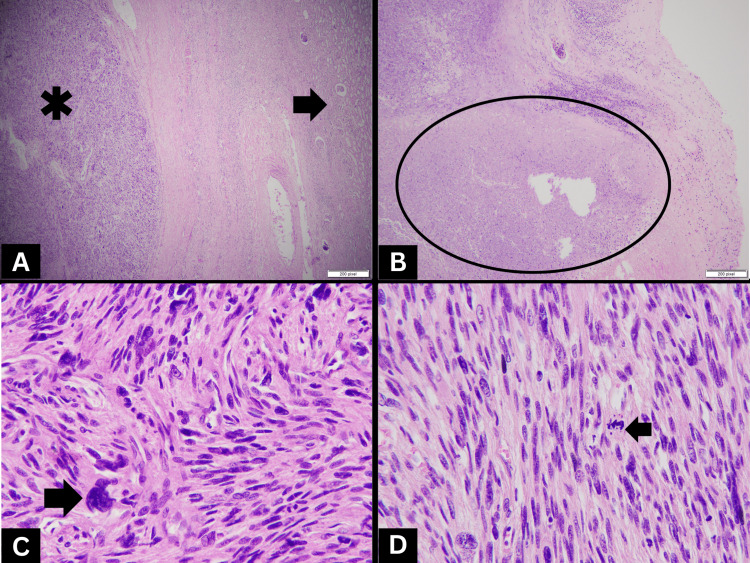
Histopathological features of renal leiomyosarcoma A): Tumor (asterisk) arranged in intersecting bundles and fascicles with adjacent renal parenchyma (arrow). B): Low-power image of tumor with areas of necrosis (circle). C): High-power image with nuclear pleomorphism. D): High-power image with atypical mitosis.

Histological examination revealed a high-grade malignant spindle-cell neoplasm arranged in interlacing bundles and fascicles (Figure 2A) along with areas of necrosis (Figure 2B). The individual cells exhibited enlarged, oval to elongated cigar-shaped nuclei with pleomorphism (Figure 2C), along with an indistinct eosinophilic cytoplasm and a few cells with bizarre nuclei. Numerous atypical mitotic figures were observed (>20/10 high-power field) (Figure 2D). No areas suggestive of other epithelial tumors were identified, even after extensive tumor sampling. No lymphovascular invasion was seen. Renal sinus fat, perinephric fat, ureteric resection margins, and adrenal gland were tumor-free.

**Figure 3:**
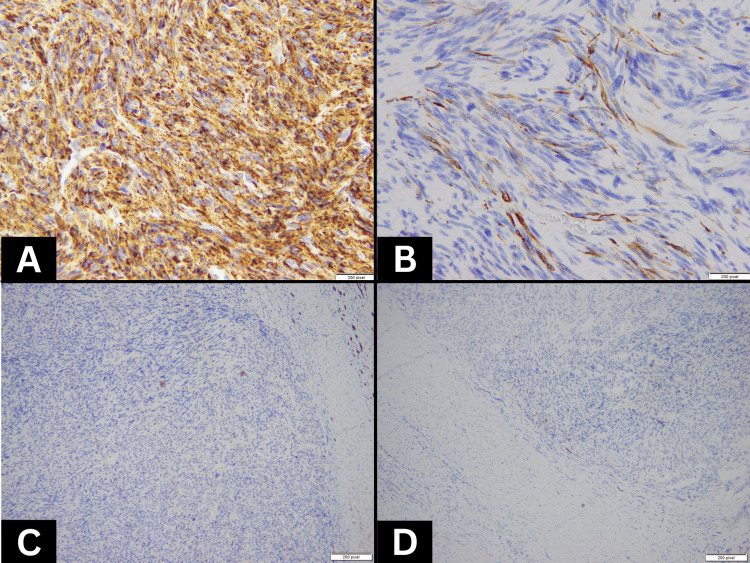
Immunohistochemical profile of renal leiomyosarcoma A): Tumor cells with strong diffuse expression for caldesmon by immunohistochemistry. B): Tumor cells with focal expression of desmin by immunohistochemistry. C): Tumor cells with no expression of cytokeratin by immunohistochemistry. D): Tumor cells with no expression of carbonic anhydrase IX by immunohistochemistry.

The immunohistochemical profile was diffusely positive for caldesmon (Figure 3A), focally positive for desmin (Figure 3B), and negative for cytokeratin (Figure 3C), confirming a diagnosis of primary renal leiomyosarcoma (Fédération Nationale des Centres de Lutte Contre le Cancer (FNCLCC) grade 3). Sarcomatoid renal cell carcinoma was ruled out by extensive sampling of the mass and negative immunostaining for cytokeratin, CD-10, and carbonic anhydrase IX (Figure 3D).

## Discussion

Primary leiomyosarcomas of the kidney is exceedingly rare, comprising only up to 1-2% of all malignant renal neoplasms [1]. Differentiating between renal cell carcinomas and leiomyosarcomas in radiology, particularly computerized tomography (CT) scans, can be challenging. Non-contrast CT scans are commonly employed for the diagnosis and staging of renal cell carcinomas. These scans reveal lesions with soft tissue attenuation ranging between 20 and 70 HU [6,7]. In the case of larger lesions, the presence of necrotic areas is frequently observed. Additionally, approximately 30% of these lesions demonstrate varying degrees of calcification [8]. CT imaging findings of leiomyosarcomas typically show heterogeneity. However, calcification is exceedingly rare in leiomyosarcoma cases [9].

A histological distinction must be made between leiomyosarcoma of the kidney and sarcomatoid renal cell carcinoma, angiomyolipoma, and leiomyoma. Both leiomyosarcomas and leiomyomas exhibit pleomorphism, but malignant tumors display necrosis and mitoses, thus allowing the distinction between the two. Bundles of smooth muscle cells and thick-walled blood vessels are interspersed with adipose tissue in renal angiomyolipomas [10]. The cytological characteristics associated with smooth muscle cells and intersecting bundles are absent in sarcomatoid carcinomas. A leiomyosarcoma has monomorphic nuclei rather than pleomorphic cells of sarcomatoid carcinomas. Sarcomatoid renal cell carcinoma is diagnosed when cytokeratin is positive in the absence of smooth muscle markers. If leiomyosarcomas express cytokeratin or epithelial membrane antigen (EMA), the desmin staining may be used in identifying leiomyosarcoma as they are positive in leiomyosarcoma, but not in sarcomatoid carcinomas [11]. In our case, there was a diffuse expression of caldesmon and focal positivity for desmin on immunohistochemistry. Sarcomas are classified by grading, which accounts for the location and extent of tumor invasion into the surrounding organs, lymph nodes, and blood vessels. This classification includes categories such as localized, regional, distant, and unstaged. However, relying solely on the extent of tumor invasion is not sufficient to evaluate prognosis because of individual factors [12]. An unfavorable prognosis is associated with leiomyosarcomas due to their aggressive behavior. With complete surgical resection, a five-year disease-free survival rate of approximately 60% is possible [13]. Prognosis in patients with renal sarcoma is not solely determined by the extent of tumor invasion, as it is influenced by a variety of factors. Lymphadenectomy remains a topic of debate but could be considered in cases with extensive tumor involvement. Prognosis is also greatly influenced by the surgical margins, as well as the histological grade. There is an approximate 90% chance of survival for low-grade tumors after five years, and approximately 30% for high-grade tumors after five years [13,14].

Localized tumors are treated with radical nephrectomy, which has a significant role to play in prognosis [15]. Although there have been reports of successful management with nephron-sparing surgery in low-grade tumors, radical nephrectomy provides superior oncologic control, particularly considering the high local recurrence rate of aggressive leiomyosarcomas [16]. As of yet, postoperative chemotherapy or radiotherapy has not been definitively proven to be effective in managing renal leiomyosarcoma [17,18]. However, adjuvant therapy is typically administered to tumors that have undergone partial resection. Promisingly, there is the possibility of exploring new treatment options such as KIT tyrosine kinase inhibitors. These inhibitors have shown efficacy in other renal tumors like renal cell carcinoma [19].

## Conclusions

To summarize, primary leiomyosarcomas of the kidney are highly uncommon and present diagnostic difficulties due to their resemblance to sarcomatoid renal cell carcinoma of the kidney. Radical nephrectomy remains the preferred treatment, and the prognosis is influenced by surgical resection and histological grade. Additional investigations are necessary to establish the potential benefits of postoperative chemotherapy or radiotherapy. The severity of the prognosis underscores the critical need for further research aimed at uncovering more effective treatment modalities for this malignancy.
